# Supplementary material for: GenomicGapID: leveraging spatial distribution of conserved genomic sites for broad-spectrum microbial identification
Source: Microbiol Spectr. 2025 Mar 14;13(5):e02817-24. doi: 10.1128/spectrum.02817-24 (PMC12054053; doi:10.1128/spectrum.02817-24)
Supplement: Supplemental material — Fig. S1 to S3; Tables S1 to S4. [file spectrum.02817-24-s0004.docx]

**Supplemental Information for**

**GenomicGapID: Leveraging Spatial Distribution of Conserved Genomic Sites for Broad-Spectrum Microbial Identification**

| **Supplementary Table 1:** Comparison of Bacterial Diagnostics to the GenomicGapID Optimized Universal Bacterial Identification System | | | | | |
| --- | --- | --- | --- | --- | --- |
|  | **Culture** | **Targeted / Multiplex PCR** | **Sequencing** | **MALDI-TOF** | **Universal Bacterial Identification System** |
| **Advantages** | Gold Standard  Hypothesis-free | Moderate Cost  Simple | Hypothesis-free  Can detect emerging pathogens | Moderate Cost  Hypothesis-free | Fast (1-2 hrs)  Cheap  Hypothesis-free  Simple |
| **Disadvantages** | Slow (12-48 hrs)  Moderate Cost  Low sensitivity  Low specificity | Hypothesis-driven  Limited to a select number of pre-defined organisms | Slow (24-120 hrs)  Expensive  Complex and labor intensive | Slow (requires culture which takes 12-48 hrs) | Currently limited to bacterial pathogens |


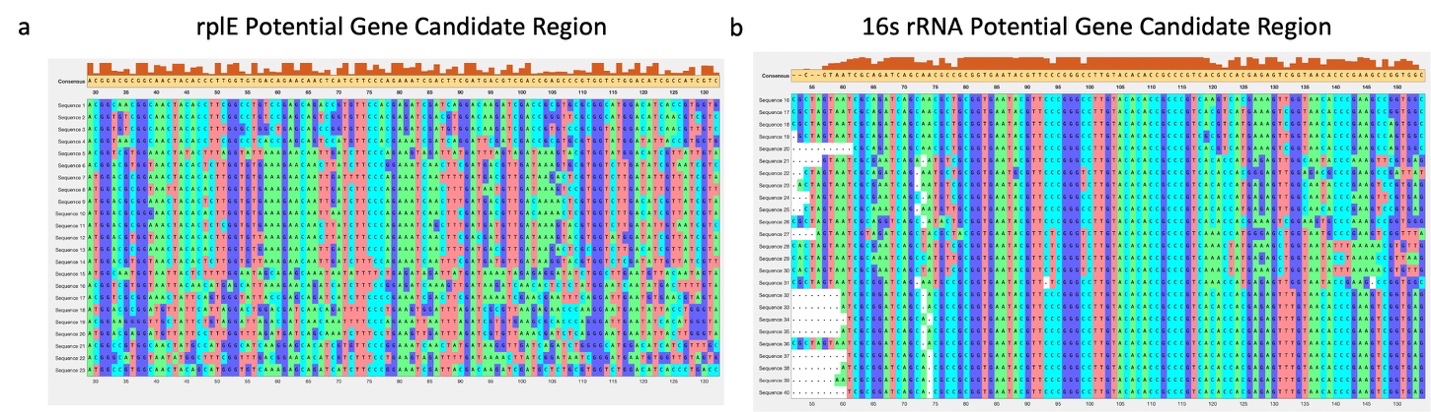


**Supplementary Figure 1. Evaluating sequence homologies of representative gene candidates.** Many of the conserved genes derived from literature do not retain sufficient sequence homology across the bacterial kingdom for primer design. As an example, (A) the rplE gene, one of the most promising gene candidates with highest coverage outside of the ribosomal genes, lacks five or more contiguous bases across its intended bacterial targets. This is in contrast to the (B) 16s rRNA gene which retains exceptional sequence conservation across the bacterial pathogen database.

Supplementary Table 2: Melt reference strand and associated primer sequences

| 200 bp reference strand | CTCTTATCACATTTGTTTCGACGACAGCCGCCTTCGCAGTTTCCTCAGACACTTAAGAATAAGCGCTTATTGTAGGCAGAGGCACGCCCTATTAGTGGCTGCGGCAAAATATCTTCGGATCCCCTTGTCCAACCAAATTGATCGAATTCTTTCATTTAAGACCCTAATATGTCATCATTAGTGATTAAATGCCACTCAAA |
| --- | --- |
| 200 bp reference strand FWD primer | CTCTTATCACATTTGTTTCGACGACAGC |
| 200 bp reference strand REV primer | TTTGAGTGGCATTTAATCACTAATGATGACATATTAGGG |

**Supplementary Table 3: Bacteria not covered by the 16s-23s universal primer pair**

| ***Borellia burgdorferi*** |
| --- |
| ***Borrelia recurrentis*** |
| ***Borrelia turicatae*** |
| ***Leptospira interrogans*** |
| ***Campylobacter lari*** |
| ***Campylobacter upsaliensis*** |
| ***Brucella canis*** |
| ***Brucella suis*** |
| ***Candidatus Liberibacter africanus*** |
| ***Candidatus Liberibacter asiaticus*** |
| ***Anaplasma phagocytophilum*** |
| ***Ehrlichia canis*** |
| ***Ehrlichia chaffeensis*** |
| ***Ehrlichia ruminantium*** |

**Supplementary Table 4: Universal primer pair sequences**

| **Primer** | **Sequence** |  |
| --- | --- | --- |
| 16s FWD primer | CTTGTACACACCGC |  |
| 23s REV primer | TCGCTCGCCACTAC |  |
| 23s FWD primer | AGAACGTCGTGAGAC |  |
| 5s REV primer | GCTTAACTTCCGGGTTCG, GCTTAACTTCTGTGTTCG, CGAGTTCGGGATGG | |
| Thr FWD primer | GCTCAGGTGGTAGAGCA |  |
| Tyr REV primer | CAGATTTACAGTCTGCC | |


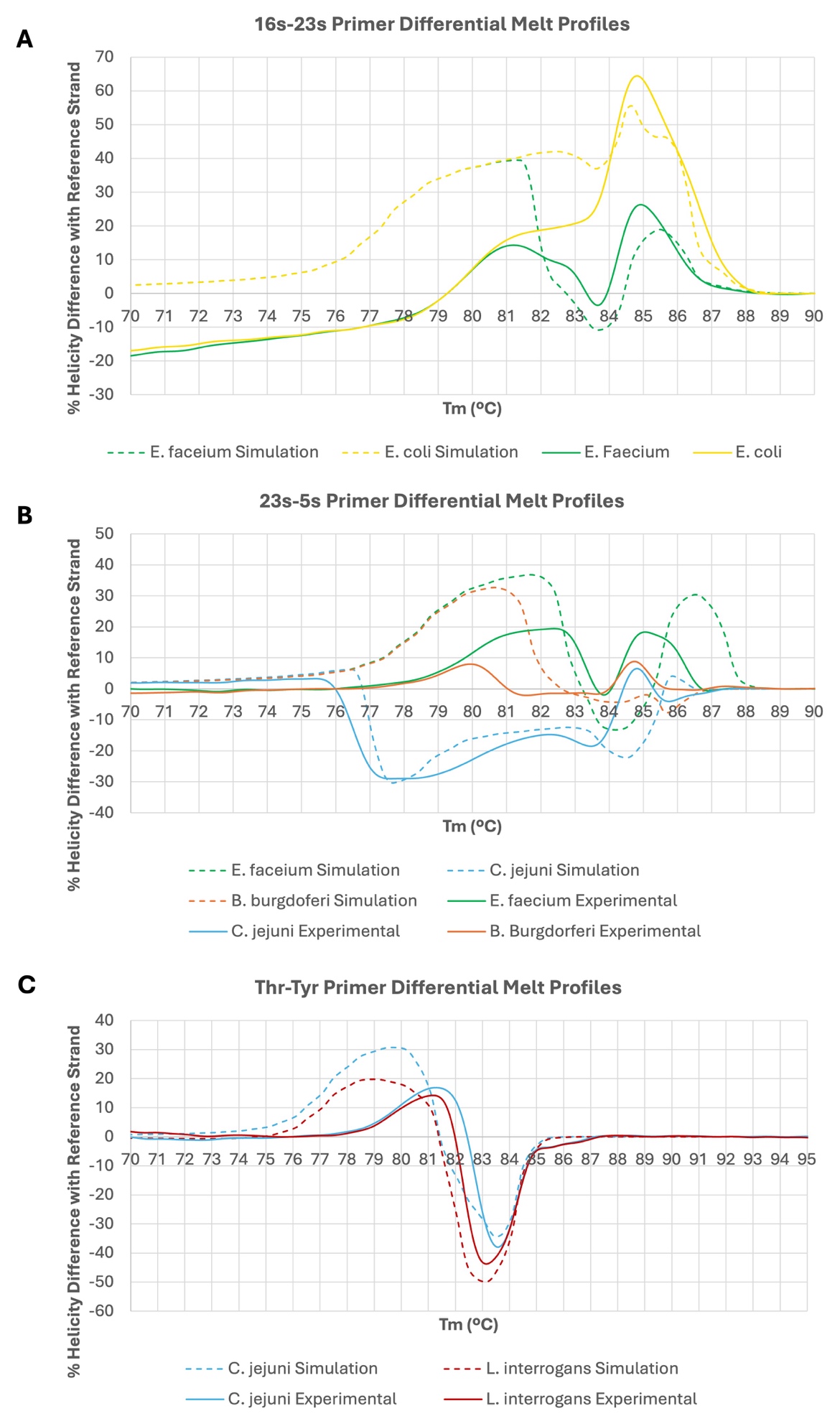


**Supplementary Figure 2. Experimental validation of universal primer pairs using melt differential.** Instead of taking the 1^st^ derivative of the melt profiles of the (A) 16s-23s, (B) 23s-5s, and (C) *thr*-*tyr* universal primer pairs as shown in Figure 5, the data can instead be visualized by taking the difference of the percent helicity between the 200 bp reference strand and target strand across all T_m_. An attempt to align simulation and experiment was made by linearly shifting each curve so that the tallest peak in the set overlapped.


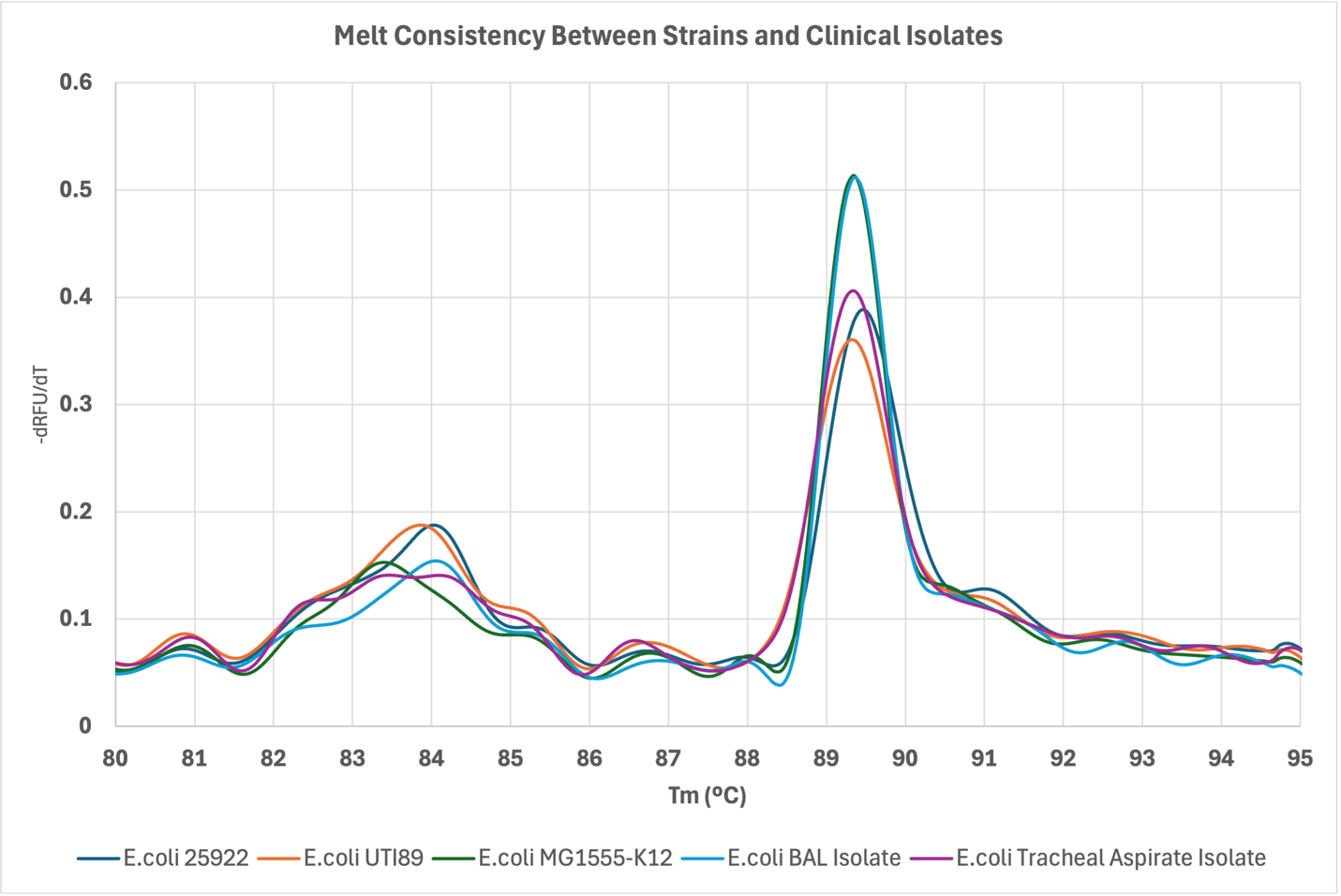


**Supplementary Figure 3. Experimental melt consistency between bacterial strains/isolates.** Varying strains and clinical isolates of *E. coli* were amplified using the 16s-23s universal primer pair. There is near complete overlap between signatures.
